# Prognostic Significance of Diastolic Dysfunction With Multiple Comorbidities in Heart Failure Patients

**DOI:** 10.7759/cureus.8297

**Published:** 2020-05-26

**Authors:** Baldeep Mann, Janpreet S Bhandohal, Savi Mushiyev

**Affiliations:** 1 Internal Medicine, Metropolitan Hospital Center, New York Medical College, New York, USA; 2 Internal Medicine, University of California, Los Angeles - Kern Medical Center, Bakersfield, USA; 3 Cardiology, Metropolitan Hospital Center, New York Medical College, New York, USA

**Keywords:** diastolic dysfunction, heart failure, readmission

## Abstract

Background

Heart failure poses a significant burden on health care and economy. In recent years, diastolic dysfunction has been increasingly recognized as a significant predictor of readmission in heart failure patients.

Objectives

We aimed to identify factors predicting readmission in patients with clinical heart failure at 30 days and six months.

Methods

A retrospective chart review was performed at a single urban medical center, including 208 patients in our final analysis.

Results

A higher Charlson comorbidity index (CCI) and moderate anemia (hemoglobin [Hb] < 10 g/dL) were significant predictors of readmission at both 30 days and six months. In addition, advanced chronic kidney disease (CKD) stage (4 or 5) and follow-up in a cardiology clinic were significant predictors at six months. During multivariate analysis, worsening diastolic dysfunction (grade 3 or 4) (OR: 2.09; 95% CI: 1.03 to 4.23), higher CCI (OR: 1.18; 95% CI: 1.03-1.36), and Hb < 10 g/dL (OR: 3.42; 95% CI: 1.44-8.13) were independent predictors of readmission at 30 days. Higher CCI (OR: 1.37; 95% CI: 1.19-1.58) and CKD stage 4 or 5 (OR: 3.05; 95% CI: 1.40-6.62) were independent predictors of readmission at six months.

Conclusions

Worse diastolic dysfunction (grade 3 or 4) was a significant predictor of all-cause readmission at 30 days post-discharge in heart failure patients. Higher CCI precisely predicted readmission as an independent variable at 30 days and six months. Anemia (Hb < 10 g/dL) and CKD stage 4 or 5 were significant predictors of readmission at 30-days and six months, respectively.

## Introduction

Heart failure (HF) affects an estimated 6.5 million Americans ≥ 20 years of age and has an expected increase in prevalence by 46% from 2012 to 2030 [1]. It is one of the leading causes of morbidity and mortality and poses a high economic burden on both the patient and the society due to high health-care-related costs projecting from $24.7 billion in 2010 to $77.7 billion in 2030 in the United States [2,3]. The incidence of HF rises sharply with advancing age, especially over 60 years [1,2]. Previously, HF was most often categorized into HF with reduced ejection fraction (HFrEF) and HF with preserved ejection fraction (HFpEF), but, currently, three categories are used based on left ventricular ejection fraction (LVEF): HFrEF (LVEF < 40%), HFpEF (LVEF ≥ 50%), and LVEF of 40%-49% categorized as mid-range (HFmrEF) [4]. Approximately 50% of patients with the clinical syndrome of HF have a preserved ejection fraction (HFpEF) in the community. Multimorbidity is common in HF but appears to be more severe in HFpEF as compared with HFrEF [5]. It has therefore become increasingly important to recognize and address diastolic dysfunction and to also determine the role of multiple comorbidities in the prognosis of patients with HF.

## Materials and methods

We aimed to study the relationship between grades of diastolic dysfunction and rehospitalization rate, as an independent factor and in combination with comorbidities, among all HF patients (including HFrEF and HFpEF) admitted to the Metropolitan Hospital Center (MHC), New York. We conducted a single-center retrospective chart review of patients aged 18 years or older with HF admitted to the center between July 1, 2010, and June 30, 2016. Patient charts were reviewed using electronic medical records. Data of patients with HFrEF (LVEF < 40%) and HFpEF (LVEF > 40%) were reviewed. First admission in MHC with symptoms or signs of HF was considered as index admission. Follow-up records were reviewed till six months from index admission to determine the number of readmissions at 30 days and six months. If a patient was admitted within 30 days, six months were calculated from the subsequent admission to eliminate duplication. Inclusion criteria were patients aged 18 years and above admitted with symptoms and signs of HF such as shortness of breath (New York Heart Association class I-IV), orthopnea and paroxysmal nocturnal dyspnea, pedal edema, elevated jugular venous distention, crepitation on lung auscultation, including both HFrEF and HFpEF, patients with baseline echocardiography to determine ejection fraction (using modified Simpson’s method) as well as the degree of diastolic dysfunction during index admission in this hospital, and patients with HF who had other labs and investigations performed such as NT-pro BNP (NT-proB-type natriuretic peptide), BUN (blood urea nitrogen), serum creatinine, hemoglobin (Hb) A1c. Serum creatinine was used to determine chronic kidney disease (CKD) staging in patients with underlying renal dysfunction [6]. Exclusion criteria include patients with HF who expired or left the hospital without echocardiography or assessment of cardiac function, patients who lost to follow-up post-hospital discharge (also including the patients who were admitted to other hospitals based on history), and patients with any terminal illness with life expectancy less than six months. In addition, patients were excluded if they had arrhythmias, paced rhythm, or poor quality of echocardiography, which would impair the determination of diastolic dysfunction. Patients who were admitted mainly for hemodialysis or ambulatory surgery were also excluded.

The primary aim of this study was to determine whether diastolic dysfunction plays a role in all-cause readmission in patients with HF. The secondary aim was to determine the burden of other comorbidities in predicting readmission. Charlson comorbidity index (CCI) was used as a criterion to determine the comorbidity burden. The study was performed in compliance with the BRANY IRB and STAR NYC-HHC protocol. Statistical analysis was performed using SPSS Version 25.0 (IBM Corp., Armonk, NY, USA). Chi-square test was used for categorical variables, and Student’s t-test was used for continuous variables. Multivariate analysis was performed using logistic regressions. A p-value of <0.05 was considered statistically significant.

## Results

**Figure 1:**
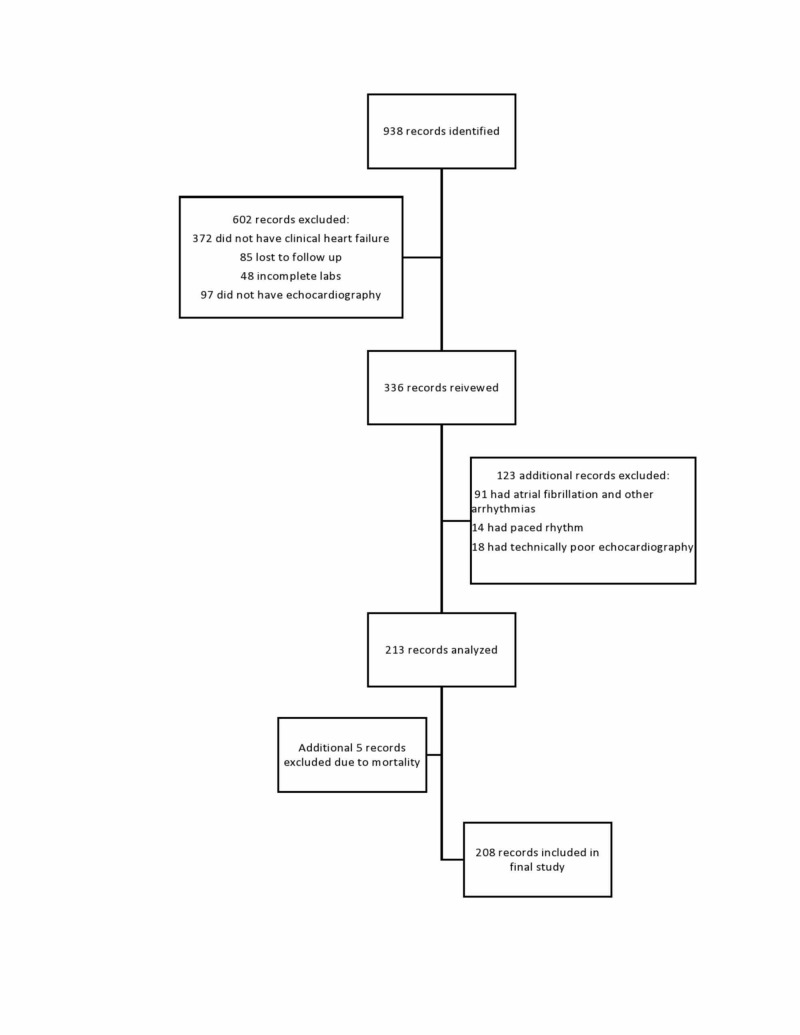
Flowchart showing the selection process of cases.

A total of 208 patients were finally included in the analysis (Figure 1). The various characteristics and factors determining readmission at 30 days and six months are given in Tables 1 and 2, respectively. Majority of our patients were males (119 [57.2%]). The mean age in the readmission group was higher than the non-readmission group at both 30 days and six months, so was the comorbidity burden as determined by CCI. CCI was reported as a continuous variable. Other variables based on laboratory results were categorized in various ways. Lower Hb was analyzed having the presence or absence of moderate anemia (Hb < 10 g/dL), troponin I was categorized as either positive or negative (>0.04 as being positive), and CKD staging was analyzed by comparing outcomes of stage 4/5 as compared to other stages (1/2/3). LVEF was categorized as either reduced or preserved (LVEF < 40% was considered reduced). The diastolic dysfunction was categorized into grades 0 to 4 based on echocardiographic criteria and was analyzed comparing grade 3 or 4 with grade 0-2 [7]. Other echocardiographic variables that were taken into account included left atrial volume index (LAVI), left ventricular end-diastolic diameter (LVEDD), left atrial diameter (LAD), and right ventricular systolic pressure (RVSP), all of them reported as continuous variables. Length of stay was another continuous variable. Patients with appropriate follow-up in the cardiology clinic (seven days post-discharge) were reported as categorical variables.

**Table 1:** Factors predicting 30-day readmission. SD, standard deviation; HTN, hypertension; Hb, hemoglobin; CKD, chronic kidney disease; DD, diastolic dysfunction; LVEF, left ventricular ejection fraction; LVEDD, left ventricular end-diastolic diameter; LAD, left atrial diameter; RVSP, right ventricular systolic pressure.

Characteristics (30-day readmission)	Yes	No	p-Value
Total	53 (25.48%)	155 (74.52%)	
Male gender	26 (49.1%)	93 (60.0%)	0.11
Age	65.28 (SD: 11.98)	63.32 (SD: 13.28)	0.342
HTN not controlled	19 (35.8%)	72 (46.5%)	0.118
Charlson comorbidity index	5.66 (SD: 2.26)	4.74 (SD: 2.29)	0.012
Hb < 10 g/dL	17 (32.1%)	19 (12.3%)	0.002
Troponin I positive (>0.04)	23 (43.4%)	77 (49.7%)	0.264
CK	169.74 (SD: 149.87)	210.37 (SD: 198.48)	0.174
HbA1c	6.99 (SD: 2.03)	7.07 (SD: 2.11)	0.817
CKD stage 4/5	15 (28.3%)	31 (20.0%)	0.144
DD grade 3/4	20 (37.7%)	42 (27.1%)	0.10
LVEF < 40%	31 (58.5%)	107 (69.0%)	0.10
LVEDD (cm)	5.54 (SD: 0.95)	5.58 (SD: 0.81)	0.757
LAD (cm)	4.33 (SD: 0.54)	4.32 (SD: 0.58)	0.962
RVSP (mm Hg)	39.00 (SD: 11.66)	37.26 (SD: 13.13)	0.393
Length of stay (days)	4.89 (SD: 4.46)	4.31 (SD: 4.06)	0.385
Seen in the cardiology clinic (7 days)	11 (20.8%)	50 (32.3%)	0.077

**Table 2:** Factors predicting six-month readmission. SD, standard deviation; HTN, hypertension; Hb, hemoglobin; CKD, chronic kidney disease; DD, diastolic dysfunction; LVEF, left ventricular ejection fraction; LVEDD, left ventricular end-diastolic diameter; LAD, left atrial diameter; RVSP, right ventricular systolic pressure.

Characteristics (six months readmission)	Yes	No	p-Value
Total	102 (49%)	106 (51%)	
Male gender	59 (57.8%)	60 (56.6%)	0.484
Age	65.27(SD: 13.34)	62.42 (SD: 12.50)	0.112
HTN not controlled	43 (42.2%)	48 (45.3%)	0.377
Charlson comorbidity index	5.65 (SD: 2.56)	4.33 (SD: 1.84)	0.000
Hb < 10 g/dL	25 (24.5%)	11 (10.4%)	0.006
Troponin I positive (>0.04)	48 (47.1%)	52 (49.1%)	0.441
CK	190.30 (SD: 156.56)	209.37 (SD: 213.93)	0.466
HbA1c	6.84 (SD: 1.79)	7.25 (SD: 2.32)	0.160
CKD stage 4/5	33 (32.4%)	13 (12.3%)	0.000
DD grade 3/4	33 (32.4%)	29 (27.4%)	0.263
LVEF < 40%	62 (60.8%)	76 (71.7%)	0.064
LVEDD (cm)	5.61 (SD: 0.84)	5.54 (SD: 0.85)	0.557
LAD (cm)	4.34 (SD: 0.52)	4.30 (SD: 0.62)	0.638
RVSP (mm Hg)	38.00 (SD: 12.25)	37.42 (SD: 13.30)	0.746
Length of stay (days)	4.96 (SD: 5.00)	3.97 (SD: 3.10)	0.087
Seen in the cardiology clinic (7 days)	24 (23.5%)	37 (34.9%)	0.049

In both 30-day readmission and six-month readmission data, higher CCI and moderate anemia (Hb < 10 g/dL) were found to be significant predictors (Table 1). In addition, advanced CKD staging (stage 4/5) and follow-up in the cardiology clinic at seven days (clinic visit being associated with lower readmission rate) were significant predictors of readmission at six months (Table 2). On multivariate analysis, worsening diastolic dysfunction, higher CCI, and decreased Hb were independent predictors of readmission at 30 days (Table 3). Higher CCI and worsening renal dysfunction were independent predictors of readmission at six months (Table 4). Diastolic dysfunction was not statistically significant at six months in predicting the readmission independently.

**Table 3:** Multivariate analysis for factors predicting 30-day readmission. OR, odds ratio; CI, confidence interval; DD, diastolic dysfunction; CKD, chronic kidney disease; Hb, hemoglobin.

Readmission (30 days)	Logistic regression adjusted OR [95% CI]	p-Value
DD grade (0-2 vs. 3/4)	2.09 [1.03-4.23]	0.041
Charlson comorbidity index	1.18 [1.03-1.36]	0.018
CKD stage 4/5	1.005 [.43-2.32]	0.99
Hb < 10 g/dL	3.42 [1.44-8.13]	0.005

**Table 4:** Multivariate analysis for factors predicting six-month readmission. OR, odds ratio; CI, confidence interval; DD, diastolic dysfunction; CKD, chronic kidney disease; Hb, hemoglobin.

Readmission (six months)	Logistic regression adjusted OR [95% CI]	p-Value
DD grade (0-2 vs. 3/4)	1.43 [0.76-2.7]	0.262
Charlson comorbidity index	1.37 [1.19-1.58]	0.001
CKD stage 4/5	3.05 [1.40-6.62]	0.005
Hb < 10 g/dL	2.187 [0.927-5.163]	0.074

LVEF was not a significant predictor for readmission. Uncontrolled hypertension, elevated troponin I, and worse HbA1c (indicating worse diabetes control) were not associated with readmission. Likewise, none of the echocardiographic parameters such as LVEDD, LAD, and RVSP were useful in predicting readmission. LAVI was not available for all the patients; therefore, a subgroup analysis was performed on 123 patients, which showed that LAVI was not statistically significant in predicting readmission independently at 30 days and six months (p = 0.88 and p = 0.99, respectively). Similarly, another subgroup analysis was performed on 178 patients who had ProBNP measured. Logistic regression showed that it was statistically significant in predicting 30-day readmission (OR: 1.0000310; 95% CI: 1.0000059-1.0000562; p = 0.015) and six-month readmission (OR: 1.000045; 95% CI: 1.00001-1.00008; p = 0.01) but given the extremely small OR would lack clinical significance.

## Discussion

There has been a paucity of data on prognosis in HF patients with diastolic dysfunction as compared with systolic dysfunction. The prevalence of diastolic dysfunction is on the upsurge, especially in the elderly, as compared with systolic dysfunction, which seems to have decreased in the 21st century [2]. In recent years, several studies have been published signifying the role of diastolic dysfunction in various groups of populations. A higher incidence of diastolic dysfunction has been found in patients with chronic obstructive pulmonary disease (COPD) [8]. It has also been associated with mechanical ventilation weaning failure, as characterized by increased E/e’ ratio [9]. Diastolic dysfunction is more common in septic patients compared with those with systolic dysfunction and has been associated with increased mortality in this group of patients, with the latter not linked to mortality [10]. Diastolic dysfunction is also associated with death, prolonged mechanical ventilation, and prolonged hospital and ICU length of stay independent of systolic dysfunction in patients undergoing cardiac surgery [11]. Perioperative diastolic dysfunction is also an independent predictor of adverse cardiovascular outcomes in patients undergoing noncardiac surgery [12]. It has also been found to be strongly associated with NAFLD (nonalcoholic fatty liver disease), a hepatic manifestation of metabolic syndrome which is on the rise in the U.S. population [13].

The quality of life, clinical symptoms, readmission rate, and six-month mortality were found to be similar in HF patients with systolic dysfunction and isolated diastolic dysfunction [14]. At the end of one year, left ventricular diastolic dysfunction was also found to be the only echocardiographic predictor of rehospitalization in survivors of acute myocardial infarction [15]. No differences were noted in mortality and readmission rate between HFrEF and HFpEF at the end of the three-year follow-up in the Japanese population [16]. It has therefore become increasingly important to interpret the status of diastolic dysfunction in patients admitted to the hospital. It can no longer be seen as an isolated cardiac dysfunction, rather it has systemic implications. Data from the OPTIMIZE-HF (Organized Program to Initiate Lifesaving Treatment in Hospitalized Patients with Heart Failure) registry revealed similar post-discharge mortality risk and equally high rates of rehospitalization in patients with HFrEF and HFpEF [17]. Restrictive filling pattern is also found to be a powerful predictor of HF hospitalization and mortality in patients post-myocardial infarction [18,19]. In another study, combining early trans-mitral flow velocity (E)/early diastolic velocity of mitral annulus (E’) with LV ejection fraction was a better predictor of readmission and cardiac death in HF patients compared with LV ejection fraction alone [20].

In our study, we found that worsening diastolic dysfunction was an independent predictor of readmission at 30 days post-discharge but not at six months after index admission for HF during multivariate analysis. These results suggest that patients with worse diastolic dysfunction were either admitted within 30 days or were not admitted even at six months. One possible explanation for this could be that the patients who had better disease control during the initial phase post-discharge were better able to maintain lifestyle modification and medication compliance so that they were not readmitted later regardless of initial diastolic dysfunction grade. On the reverse, non-compliance with medications and follow-up might have played a role for 30-day readmission in patients with worse diastolic dysfunction. Although used to determine diastolic dysfunction wherever available, left atrial volume index was not a significant predictor independently. LVEF was also not a significant predictor for readmission. Patients who were not admitted initially, during the six-month follow-up, comorbidities played a major role in readmission, especially renal dysfunction. These findings are similar to what Miyagishima et al. have found [16]. They also concluded that the presence or absence of reduced LVEF was not useful in predicting the prognosis, and, on the other hand, anemia and renal dysfunction were identified as significant prognostic factors [16]. Anemia has been found to be associated with increased long-term mortality rate in patients with diastolic HF [21]. Lower Hb along with other comorbidities such as diabetes, eGFR (estimated glomerular filtration rate), and lower blood pressure were associated with poor outcomes in patients with HFpEF [22]. In this study, Hb of less than 10 g/dL was associated with higher readmission at 30 days and six months but was only an independent predictor at 30 days. One might consider severe anemia with/without bleeding as a predisposing factor for early readmission. Due to the extremely small OR for ProBNP, an ROC (receiver operating characteristic) analysis was performed. It suggested a link between the categorical value of ProBNP greater than 3,577 for 30-day readmission and greater than 7,540 for six-month readmission. These cutoff values were statistically significant during regression analysis in predicting readmission (OR: 3.38, 95% CI: 1.58-7.24, p = 0.001, specificity = 0.5227, sensitivity = 0.7556 for 30 days; and OR: 3.40, 95% CI: 1.73-6.69, p = 0.0004, specificity = 0.8068, and sensitivity = 0.4494 for six months). Whether these threshold values have any clinical significance needs to be further evaluated in future studies.

The most significant predictors for readmission in our study at 30 days and six months were higher Charlson comorbidity index and worse renal dysfunction (CKD stage 4 and 5). Similar findings were seen in a study conducted on national database concluded that higher comorbidity burden as determined by CCI in patients of HF with diastolic dysfunction predicted higher readmission rate at 30 days, as was the renal failure [23]. Similar results were found in a large number of COPD patients in whom CCI provided meaningful prediction for readmission at 30 days [24]. Another large-scale study conducted on the Chinese population determined that higher CCI was independently associated with a risk of 30-day readmission in dialysis patients (both hemodialysis and peritoneal dialysis) [25]. CCI has also been found to be an important determinant for length of stay, mortality, and rehospitalization in the elderly [26].

Our study has a few limitations worth mentioning. First, it is a retrospective chart review and a relatively small-sized study conducted at a single center. Our hospital mainly caters to Hispanic and African-American population, which might compromise the generalizability to other patient populations. Secondly, the sample size is relatively small, possibly resulting in statistically non-significant results in certain outcomes such as length of stay, which might otherwise become significant with a bigger sample size. Third, patient follow-up was limited to six months; having a longer follow-up would better determine the long-term implications of worse diastolic dysfunction and comorbidities. Fourth, other variables such as psychosocial issues and insured versus uninsured were not included as part of variables.

## Conclusions

In our study, we concluded that worse diastolic dysfunction (grade 3 or 4) independently predicted all-cause readmission at 30 days post-discharge in HF patients. CCI precisely predicted readmission as an independent variable at 30 days and six months. Anemia (Hb < 10 g/dL) and CKD stage 4 or 5 were significant predictors of readmission at 30 days and six months, respectively. LVEF was not a significant predictor of readmission.
